# Supplementary material for: Association between occupational testicular radiation exposure and lower male sex ratio of offspring among orthopedic surgeons
Source: PLoS One. 2021 Dec 31;16(12):e0262089. doi: 10.1371/journal.pone.0262089 (PMC8719764; doi:10.1371/journal.pone.0262089)
Supplement: Attachment — Submitted filename: Response_to_Reviewers.docx [file pone.0262089.s002.docx]

**Response to ‘Journal Requirements’ :**

**1. Please ensure that your manuscript meets PLOS ONE's style requirements, including those for file naming. The PLOS ONE style templates can be found at**

[**https://journals.plos.org/plosone/s/file?id=wjVg/PLOSOne_formatting_sample_main_body.pdf**](https://journals.plos.org/plosone/s/file?id=wjVg/PLOSOne_formatting_sample_main_body.pdf)

**and**

[**https://journals.plos.org/plosone/s/file?id=ba62/PLOSOne_formatting_sample_title_authors_affiliations.pdf**](https://journals.plos.org/plosone/s/file?id=ba62/PLOSOne_formatting_sample_title_authors_affiliations.pdf)

Response

Thank you for the materials. We have referred to them again and carefully checked that the revised manuscript conforms to the style of your journal.

**2. In your ethics statement in the Methods section and in the online submission form, please ensure that you have discussed whether any identifying information was collected in the questionnaire, or whether the researchers had contact with the respondents.**

Response

Thank you for this important remark. The researchers did not collect data. They were provided data from each hospital in an anonymized form. The method of data collection has been added (lines 92-94, page 5 of the 'Manuscript').

**3. Please include your actual numerical p-values in Table 3.**

Response

As instructed, we have added the actual numerical *p* values in Table 3.

**4. In your Methods section, please provide additional information about the participant recruitment method and the demographic details of your participants. Please ensure you have provided sufficient details to replicate the analyses such as:**

**a) the recruitment date range (month and year),**

**b) the names of the five institutions that participated,**

**c) a table of relevant demographic details or doctors if available,**

**d) a statement as to whether your sample can be considered representative of a larger population, and**

**e) a description of how participants were recruited.**

Response

Thank you for these suggestions. We have responded to each item below.

a) The date of the survey has been added (line 89 , page 5 of the 'Manuscript').
b) The names of the five institutions have been added (lines 84-86, page 5 of the 'Manuscript').
c) The following table could have been inserted, but it was not included in the manuscript since it has little information that is contributive to the objective of this study. If you would like us to include it please let us know.

| Paternal department | Total | Number of females |
| --- | --- | --- |
| **RT group (n=27)** |  |  |
| General Orthopedics | 22 | 15 (68) |
| Spine Surgery | 5 | 4 (80) |
| **RNT group (n=30)** |  |  |
| Neurosurgery | 17 | 7 (41) |
| Cardiovascular medicine | 13 | 4 (31) |
| **N group (n=52)** |  |  |
| Thoracic surgery | 8 | 6 (75) |
| Cardiovascular surgery | 8 | 2 (25) |
| General Surgery | 7 | 4 (57) |
| Internal medicine | 6 | 2 (33) |
| Anesthesiology | 5 | 0 (0) |
| Resident | 4 | 0 (0) |
| Urology | 3 | 2 (67) |
| Radiology | 3 | 1 (33) |
| Emergency Department | 2 | 0 (0) |
| Pediatrics | 2 | 0 (0) |
| Ophthalmology | 1 | 1 (100) |
| Gynecology | 1 | 1 (100) |
| Medical student | 1 | 0 (0) |
| Postgraduate student | 1 | 0 (0) |
| N, departments that did not use medical radiation, including medical students; RT, departments that used medical radiation and had a high possibility of testicular radiation exposure; RNT, departments that used medical radiation but had a low possibility of testicular radiation exposure. Data are presented numbers (%). | | |

d) Like typical private hospitals in Japan, there is nothing special about this research setting. In addition, since we received responses from all the subjects; we believe that the sample was representative of the research setting. However, these hospitals are located in the Kyushu region of Japan and are not geographically representative of Japan. We have not included this information in the limitation due to concerns about redundancy, but will do so if necessary.

e) We described how we recruited participants and obtained their consent to participate (lines 92-95, page 5 of the 'Manuscript').
**Response to Reviewers:**

**Reviewer #1**

Thank you for your important remarks. Our responses to your comments are found below.

**GENERAL COMMENTS TO THE AUTHORS:**

**This paper describes the results of a survey of full-time male doctors employed in 3 different professions with access to radiation with regards to the sex of their 109 children. The doctors responded to a survey asking questions about work 1 year ahead of child’s birth. The authors speculate that inadequate protection from x-ray radiation from fluoroscopy during orthopedic surgery could have an effect on the sex ratio of children born after radiation exposure. This conclusion is not supported by the data and a giant leap is made from a small survey with absolutely no information on the nature and magnitude of radiation exposures to the conclusion that “results imply an association between testicular radiation exposure and low male sex ratio of offspring. This result might help young orthopedic surgeons to recognize the risks of radiation exposure and to take protective action against it.”**

**There are multiple factors that could affect the ratio of boys to girls and the biological mechanism for this association has not been proposed in this paper. Before any conclusions are made, it would be necessary to understand the magnitude of radiation exposures in this group of physicians and the amount of scatter radiation to the testes from fluoroscopic procedures during orthopedic surgery.**

Response

Thank you for your valuable suggestions. As you pointed out, the sample size of this study is small, and the methods are not rigorous; therefore, we understand that we can only report only exploratory results. We have improved the content of the manuscript (particularly the interpretation of the results) and included a review of the existing evidence. We would love to hear from you concerning this.

**SPECIFIC COMMENTS TO THE AUTHORS:**

**INTRODUCTION**

**Line 60:**

**“[8-13].”: Ref #12 is not appropriate.**

Response

We read the study by Ashore (2010) and realized that there was a problem with the Canadian NDR. Ref #12, Sent (2001), has been deleted as it was cited for background information only and does not relate significantly to the content of the manuscript. Thank you very much for providing the reference. It was very helpful.

**Line 67:**

**“sex ratio of the offspring”: The authors need to cite literature (if exist) that this concern is justified.**

Response

Thank you for this plausible suggestion. The paper that prompted us to conduct this study is Zadeh HG (1997), modified Ref #16 (Ref. #17 before revision). This reference has been cited in the manuscript (lines 72-74, page 4 of the 'Manuscript'), and its contents have been presented and compared with the results of the present study in the *Discussion* (lines 219-221, page 12 of the 'Manuscript').

**MATERIALS AND METHODS**

**Line 81:**

**“a retrospective longitudinal analysis”: Should use standard epidemiological study designs, e.g. cohort study.**

Response

Thank you for your suggestion. Your comment on recall bias below helped us to understand that this was a case-control design. We have changed longitudinal analysis to case-control study (line 83, page 5 of the 'Manuscript').

**Line 87:**

**“a questionnaire survey”: I am not sure I agree. Even questionnaires require consent. Do you have an IRB approval for this study? After reaching the paper through, I think it is required.**

Response

Thank you for your question. On June 8, 2021, an additional ethical review was carried out by the certified ethics committee of the main research institution, Shinkomonji hospital, regarding the ethical appropriateness of the analysis and publication of the results using the dataset currently in our possession. The study was approved. We have added this information in the *study design and setting* (lines 90-91, page 5 of the 'Manuscript').

**Line 100:**

**“recall bias”: Recall bias only happens in case-control studies; therefore, this cannot be a longitudinal study.**

Response

Thank you for this remark. We recognized that a case-control design was most appropriate, as the study measured the presence or absence of the outcome (female sex of the child) at the time of the survey and measured the presence or absence of the exposure (opportunity for testicular radiation exposure) retrospectively. As mentioned above, we have revised the study design section (line 83, page 5 of the 'Manuscript').

**Line 119:**

**“lateral and posterior radiation exposure.”: How much of the radiation dose could be received this way? Could you please give us an idea? What is the mean cumulative, badge-based dose for study participants? What percentage of it do you think could be due to scatter radiation.**

Response

Thank you for these very important questions. As we mentioned in the *Discussion*, we are aware that one serious limitation of this study was the inability to measure the actual radiation dose. Since we were unable to provide additional data, we cited the study by Funao et al. (modified ref. 18), wherein the radiation dose was similar to ours (lines 131-136, page 7 of the 'Manuscript').

**DISCUSSION**

**Line 181:**

**“comprehensive”: Why does this study qualify to be called “comprehensive”?**

Response

Thank you for your question. We referred to the study as ‘comprehensive,’ because we had data for the entire cohort, as all subjects in the study settings were included. However, following your remarks, we understood that the study is a case-control study. We have deleted the term ‘comprehensive’ since it is not appropriate in a case-control design i.e., the source cohort is not captured (lines 198, page 11 of the 'Manuscript').

**Line 187:**

**“who are exposed”: If this is correct, there are bigger concerns about the effects of radiation in those physicians than changing sex ratio.**

Response

Thank you for pointing this out. The phrase ‘who are exposed’ was clearly an exaggeration. We have replaced it with a more conservative term, ‘who might be exposed.’ There is no doubt that inexperienced doctors (as we were) tend to rely more on radiography in clinical practice, and the results of ref.5 suggest this. To confirm that the younger generation is more exposed to radiation, radiation exposure should be measured. The Society for MIST (<https://s-f-mist.com/en/>), of which we are members, has taken the lead in conducting a survey to determine the extent to which radiation damage is actually occurring; publication of the results is pending.

**Line 193:**

**“89%”: What was the response rate in this study?**

Response

Thank you for your question. Although the sample size was small, the response rate was 100%. It has been stated in the *Results* that ‘the responses were obtained from all eligible doctors.’ We have also added the percentage of responses (lines 170, page 9 of the 'Manuscript').

**Line 246:**

**“imply a chance effect.”: What were the badge doses?**

Response

Thank you for your question. We could not obtain information on the actual radiation dose around the testes for each group, as the rate of badge wearing was very low in the target population, and the badges were never worn around the testes. The term ‘chance effect’ was used because we could not explain the mechanism by which the sex ratio skewed towards males in the RNT and RT groups. Therefore, it was most likely a ‘chance effect.’

**CONCLUSIONS**

**Line 252:**

**This is a case-control study: you started with cases (male offspring) and controls (female offspring) and then interviewed them about place of work 1 year before child’s birth. However, because outcomes are not rare (~50%), odds do not approximate risks. This is most likely due to biased sample selection and recall bias.**
Response

Thank you for these important remarks. As you pointed out, this is a case-control study, and the outcomes are not rare. Therefore, the odds ratio cannot be interpreted as a risk ratio. This means that therefore, we cannot conclude from the results that ‘the RT group was four times more likely to have daughters.’ However, we believe that the odds ratios were useful in determining whether there was an association. We also believe that there was no significant selection bias, at least in the target population, as all respondents were available at the time of the survey. Furthermore, although participants had to recall information from up to 11 years prior to the study, it was easy for them to recall their department at that time; therefore, the recall bias was small.

The manuscript has been revised substantially to bring it closer to a reasonable interpretation. First, the *Key results and clinical implications* were revised for a better interpretation of the results (lines 198-208, page 11-12 of the 'Manuscript'). Second, to avoid misunderstanding about the odds ratios, we have added a limitation to the *Strengths and limitations* section (lines 289-293, page 15 of the 'Manuscript'). Finally, the claims made in the *Conclusions* have been modified (lines 51, page 3 and, lines 303-304, page 16 of the 'Manuscript').

**REFERENCES**

**Line 312:**

**“12.”: It is hard to understand why this reference was chosen. See Ashmore et. al. 2010, which addresses problems with Canadian NDR.**

Response

Ref #12 has been deleted. Thank you for pointing this out.

**Reviewer #2**

Thank you for your valuable suggestions that have helped us improve our manuscript. Our responses to each of your comments are presented below.

**General comments**

**This is an interesting and generally well written examination of sex ratio in offspring of orthopedic surgeons. The findings, although apparently quite strong, are somewhat outweighed by the absence of quantitative dose information. The authors use of the 12 months preceding birth is an odd one. If the sex ratio changes are a spermatid effect then the relevant period is from 12 to 9 months before the birth of a child, while if the changes are a spermatogonial effect than then anything prior to 12 months before birth should be considered. The Discussion is a little skimpy, and the “Comparison with other studies” section could usefully consider the findings in other studies, which are mostly null or in the opposite direction to the present study. I think the authors should tone down the conclusions in the Abstract and at the end of the Discussion in the light of the problems with this study and the lack of support of their findings from other studies. Although the MS is generally clear and well written, the language is occasionally non-idiomatic and would benefit from services of a native English speaker.**

Response

Thank you very much for summarizing the results and highlighting the issues. We will respond to your comments in more detail below. This first submitted version of the manuscript was edited by Editage (<https://www.editage.jp/>). We sent it to Editage for proofreading again.

**Detailed comments (page, line)**

**p.3 l.48-49 This sentence (“This result …”) should probably be toned down in the light of the problems with this study and the lack of support of these findings from other studies.**

Response

Thank you for pointing this out. The interpretation of our results was exaggerated. We have substantially revised the *Key results and clinical implications* (lines 198-208, page 11-12 of the 'Manuscript') and *Conclusions* (lines 51, page 3 and lines 303-304, page 16 of the 'Manuscript') to understate the interpretation of the results.

**p.7 l.130-137 The use of one year before the birth of the child as the relevant period is odd. If the sex ratio changes are a spermatid effect, as the authors seem to hypothesize then the relevant period is from 12 to 9 months before birth (i.e. 3 months before conception), since the process of sperm maturation is about 2-3 months. If spermatogonial effects are more relevant then it would be anything before 12 months before birth (i.e., more than 3 months before conception). The analysis needs to be changed to reflect these biological data.**

Response

Thank you very much for your valuable comments. As you pointed out, we stated the rationale for using the one year cut-off poorly, and the inaccuracy of the rationale was not fully explained. We hypothesized that the period at risk was the period after the secondary spermatocyte formation, when the sperm carries either the X or Y chromosome. AS you pointed out, the period at risk is not a time point, but a period with a range of 2–3 months (the period during which the secondary spermatocyte becomes sperm and is stored in the epididymis). Therefore, limiting the measurement of an exposure to a single time point may lead to misclassification. However, we believe that the magnitude of misclassification was small because it is rare for a personnel’s department affiliation to be changed within 2–3 months. Furthermore, limiting the exposure measurement to ‘one year ago’ makes it easier to be explained. Most parent do not forgets their child's birthday; therefore, we thought that it would be very easy to answer the question: ‘What department were you in exactly one year before your child's birthday?.’ We are unsure whether this was responsible for the 100% response rate.

We have added more accurate information about spermatogenesis in the *Outcome of interest and exposure* section (lines 143-153,　page 7-8 of the 'Manuscript'). In addition, the possibility of a misclassification of the exposure has been added to the *limitations* (lines 280-284, page 15 of the 'Manuscript').

**p.11 l.190-p.12 l.205 The statement “The results of this study are consistent with the available evidence” is not altogether correct. The study of Jablon & Kato (Am J Epidemiol 1971 93 253-8) of the offspring of Japanese atomic bomb survivors found no paternal-radiation-associated change in sex ratio. Dickinson et al (J Epidemiol Commun Health 1996 50 645-52) found some evidence of elevation of male:female sex ratio in offspring of British nuclear workers receiving >10 mSv 90-day preconceptional dose (so in the opposite direction to that implied by this study) but there was no association with total preconceptional dose. Shea et al (Am J Epidemiol 1997 145 546-51) studied a general UK population and found that the sex ratio in exposed fathers was the same as in unexposed fathers; however, there was no dosimetry in this study. Koya et al (Radiat Environ Biophys 2015 54 453-63) observed no change in sex ratio with father’s preconceptional dose in the Kerala high background radiation area of India. Scherb et al (Environ Health 2013 12 63) document a jump in the sex ratio, with increased proportion of male births, post 1986 in Russia and Cuba, which they attribute to radioactive contamination from the Chernobyl nuclear accident; however there was no dosimetry in this study, but nevertheless the results point in the opposite direction to those in the present study. The study of Hama et al that the authors discuss is interesting, but it is based on a self-selected survey sample and the possibility of selection bias cannot be discounted. The very small reduction in sex ratio in the study of Pillarisetti et al is probably not significant. The Discussion section could usefully discuss all these findings.**

Response

Thank you very much for all the reference-by-reference information you have provided. In the manuscript, we only cited previous studies on occupational exposure to medical X-rays among healthcare workers. We have reviewed the papers you suggested and have made significant additions and revisions to the *Comparison with other studies* section for a more in-depth discussion (lines 228-247, page 13 of the 'Manuscript').

**p.13 l.233-234 This sentence (“First, exposure variables … exposure.”) is somewhat misleading. Quantitative radiation exposure variables are not available in the present study, or at least not given. The only measure of exposure is the type of job performed, which is probably a poor surrogate. It might be best if this is rephrased as “First, the fact of possible radiation exposure may be misclassified, and there is no measurement of actual testicular radiation exposure.”**

Response

Thank you for your detailed remarks. We have revised the sentence (lines 275-277, page 15 of the 'Manuscript').

**p.13 l.238-239 The sentence “Therefore it is not possible…” should probably be removed, as it can be inferred from what is said previously, assuming the sentence above is modified in the say suggested.**

Response

We have deleted the sentence as suggested.

**p.14 l.254-255 This sentence (“This result …”) should probably be toned down in the light of the problems with this study and the lack of support of these findings from other studies.**

Response

Following your remarks, we have toned down our conclusions. Thank you very much for reading the manuscript in detail and for your constructive comments. We have revised in the *Conclusions* (lines 51, page 3 and, lines 303-304, page 16 of the 'Manuscript').
