# Supplementary material for: Association between occupational testicular radiation exposure and lower male sex ratio of offspring among orthopedic surgeons
Source: PLoS One. 2021 Dec 31;16(12):e0262089. doi: 10.1371/journal.pone.0262089 (PMC8719764; doi:10.1371/journal.pone.0262089)
Supplement: Attachment — Submitted filename: Hijikata et al PLoS ONE re-review 2021.docx [file pone.0262089.s003.docx]

**Comments to the authors on “Association between occupational testicular radiation exposure and lower male sex ratio of offspring among orthopedic surgeons” by Y. Hijikata, M. Nakahara, A. Kusumugi, J. Morii, N. Okubo, N. Hatano and Y. Takahashi (PONE-D-21-09546R1)**

**General comments**

This is an interesting and generally well written examination of sex ratio in offspring of orthopedic surgeons. The paper is much improved on the first version. However, there are still quite a few weaknesses, both in the presentation of the Tables and particularly in the Discussion.

**Detailed comments (page, line)**

p.3 l.51	I would replace the final sentence by “Confirmatory evidence is needed from larger studies which measure the pre-conceptional doses accumulated in various temporal periods, separating out spermatogonial and spermatid effects.”.

p.8 l.146-153	What evidence is there in support of this hypothesis, that sex ratio results entirely from spermatid effects? If this is (as I suspect) a largely *post hoc* hypothesis then this should be made clear.

p.8 l.148	I assume that by “2-3 months” is meant “2-3 months before conception”.

p.8 l.151-153	Is this sentence (“This means that…”) correct? Surely it is the period 1-3 months before conception rather than the date of birth that is meant.

Tables 1, 2, 3	It is needlessly confusing that in Table 1 the proportion of female children is given, whereas in Table 2 it is effectively the proportion of male children that is given. Although the labelling of Table 3 does not make this clear, it must be the case that it is the odds ratio of female:male births that is given. The labelling of Table 3 must be improved, and all three Tables use more or less the same measure (males/total or females/total, it does not matter which).

p.12 l.211 – p.13 l.227	The sentence on p.12 l.211-212 (“The results of this study are consistent …”) should be removed or at least toned down. The evidence cited in support of this statement in the rest of the para is really rather weak. As previously noted by the referee the study of Hama *et al* that the authors discuss is interesting, but it is based on a self-selected survey sample and the possibility of selection bias cannot be discounted. The very small reduction in sex ratio in the study of Pillarisetti *et al* is probably not significant. These weaknesses and limitations of this evidence must be discussed here.

p.13 l.224-227	This sentence (“The results of this study, obtained …”) repeats what is said at the beginning of the Discussion, and should be removed.

p.13 l.246-247	This sentence (“Therefore, it should be noted …”) is rather odd. Are the authors really implying that the effects observed are not due to the radiation alone, but to some other occupational factor specific to healthcare workers? Obviously if the effects are related to radiation exposure (rather than some unknown factor specific to this occupational group) there is no reason why they should not apply to all radiation exposed groups. If the findings relate to some factor specific to this occupational group then this considerably lessens their scientific interest, and begs the question as to whether radiation exposure is really the cause of what has been observed in this study.

p.15 l.276-277	There should be discussion at this point of the limitations of the data in relation to timing of exposure. In particular the data make it quite difficult to differentiate between spermatid effects (which the authors assume, as above, but possibly based on no strong prior body of data) and spermatogonial effects. This hypothesis could also be discussed.

p.15 l.293 – p.16 l.294	The sentence is not terribly clear, and the authors should also highlight the role that confounding by some unknown factor may play. So perhaps rephrase as: “Finally, although the results were highly statistically significant, they are based on a quite small sample, and the play of chance, or some unidentified confounding factor, cannot be ruled out.”.

p.16 l.303-304	I would replace the final sentence by “Confirmatory evidence is needed from larger studies which measure the pre-conceptional doses accumulated in various temporal periods, separating out spermatogonial and spermatid effects.”.

**Comments to the editors on “Association between occupational testicular radiation exposure and lower male sex ratio of offspring among orthopedic surgeons” by Y. Hijikata, M. Nakahara, A. Kusumugi, J. Morii, N. Okubo, N. Hatano and Y. Takahashi (PONE-D-21-09546R1)**

This is an interesting and generally well written examination of sex ratio in offspring of orthopedic surgeons. The paper is much improved on the first version. However, there are still quite a few weaknesses, both in the presentation of the Tables and particularly in the Discussion. These problems are however entirely fixable, and were they to be amended the paper should be considered for publication.

Mark Little

Radiation Epidemiology Branch

National Cancer Institute

8^th^ August 2021
