# Supplementary material for: Association between occupational testicular radiation exposure and lower male sex ratio of offspring among orthopedic surgeons
Source: PLoS One. 2021 Dec 31;16(12):e0262089. doi: 10.1371/journal.pone.0262089 (PMC8719764; doi:10.1371/journal.pone.0262089)
Supplement: Attachment — Submitted filename: Response_to_Reviewers.docx [file pone.0262089.s004.docx]

**Response to Reviewers:**

**Reviewer #2**

**General comments**

**This is an interesting and generally well written examination of sex ratio in offspring of orthopedic surgeons. The paper is much improved on the first version. However, there are still quite a few weaknesses, both in the presentation of the Tables and particularly in the Discussion.**

Response: Thank you for your insightful comments. We have revised the manuscript in line with your suggestions. We hope that our edits and the responses we have provided below satisfactorily address all the issues and concerns you have noted and that the revised manuscript is now suitable for publication.

**Detailed comments (page, line)**

**p.3 l.51**

**I would replace the final sentence by “****Confirmatory evidence is needed from larger studies which measure the pre-conceptional doses accumulated in various temporal periods, separating out spermatogonial and spermatid effects.”.**

Response: Thank you for this comment. We have accordingly replaced the final sentence (p.3 l.47-49).

Revise: Confirmatory evidence is needed from larger studies which measure the pre-conceptional doses accumulated in various temporal periods, separating out spermatogonial and spermatid effects.

**p.8 l.146-153**

**What evidence is there in support of this hypothesis, that sex ratio results entirely from spermatid effects? If this is (as I suspect) a largely post hoc hypothesis then this should be made clear.**

Response: Thank you for your comment. To clarify, this is not a post-hoc hypothesis. There is a widespread rumor among orthopeadic surgeons in Japan that the children of orthopedic surgeons tend to be female. If this is indeed true, we would like to understand why this is so. Thus, we formulated this hypothesis and designed this study to test it. However, as you have pointed out, this hypothesis has no supportive evidence, and we have accordingly made this clear (p.7 l.141).

Revise: We hypothesized, albeit without supporting evidence, that…

**p.8 l.148**

**I assume that by “2-3 months” is meant “2-3 months before conception”.**

Response: Yes, and per your comment, we have revised this in the manuscript for clarity (p.8 l.143-144).

Revise: 2–3 months before conception.

**p.8 l.151-153**

**Is this sentence (“This means that…”) correct? Surely it is the period 1-3 months before conception rather than the date of birth that is meant.**

Response: We apologize for the unclear text. We have now corrected this to "The risk period began one year before the child’s birthday” (p.8 l.146-149).

Revise: In other words, considering that the gestation period is approximately 280 days, the period at risk of a decrease in the male sex ratio of the child due to testicular radiation exposure began approximately one year before the child's birthday.

**Tables 1, 2, 3**

**It is needlessly confusing that in Table 1 the proportion of female children is given, whereas in Table 2 it is effectively the proportion of male children that is given. Although the labelling of Table 3 does not make this clear, it must be the case that it is the odds ratio of female:male births that is given. The labelling of Table 3 must be improved, and all three Tables use more or less the same measure (males/total or females/total, it does not matter which).**

Response: Thank you very much for your meaningful comments. We apologize for the confusing presentation of our tables. Per your comment, we have presented all calculated sex ratios in this study as female sex ratio and have accordingly revised the relevant sections (i.e., *Abstract* [p.2 l.33-34 and l.41], *Mateials and Methods* [p.8 l.155-156], *Results* [p.10 l.183, l.185], and Tables 1 and 2). Moreover, in other instances, we have clearly indicated whether we are referring to female or male sex ratio (p.4 l.70, p.5 l.75, p.7 l.139, l.142, p.8 l.148 and l.155). We have also revised the title of Table 3 to clearly indicate that the odds ratios of having female children are presented (p.11 l.207).

**p.12 l.211 – p.13 l.227**

**The sentence on p.12 l.211-212 (“The results of this study are consistent …”) should be removed or at least toned down. The evidence cited in support of this statement in the rest of the para is really rather weak. As previously noted by the referee the study of Hama et al that the authors discuss is interesting, but it is based on a self-selected survey sample and the possibility of selection bias cannot be discounted. The very small reduction in sex ratio in the study of Pillarisetti et al is probably not significant. These weaknesses and limitations of this evidence must be discussed here.**

Response: Thank you for your significant remarks. We have revised the tone of this particular sentence (p.12 l.234) and corrected the last sentence of the paragraph (p.13 l.245-246).

Revise: Our results are consistent with the inconclusive evidence…

However, these results are limited by selection biases because they used questionnaires with voluntary responses.

**p.13 l.224-227**

**This sentence (“The results of this study, obtained …”) repeats what is said at the beginning of the Discussion, and should be removed.**

Response: We have deleted this sentence per your comment.

**p.13 l.246-247**

**This sentence (“Therefore, it should be noted …”) is rather odd. Are the authors really implying that the effects observed are not due to the radiation alone, but to some other occupational factor specific to healthcare workers? Obviously if the effects are related to radiation exposure (rather than some unknown factor specific to this occupational group) there is no reason why they should not apply to all radiation exposed groups. If the findings relate to some factor specific to this occupational group then this considerably lessens their scientific interest, and begs the question as to whether radiation exposure is really the cause of what has been observed in this study.**

Response: Thank you for pointing this out. We believe that radiation affects not only medical professionals but also the general population. We understand that our phrasing was misleading and have deleted the sentence accordingly.

**p.15 l.276-277**

**There should be discussion at this point of the limitations of the data in relation to timing of exposure. In particular the data make it quite difficult to differentiate between spermatid effects (which the authors assume, as above, but possibly based on no strong prior body of data) and spermatogonial effects. This hypothesis could also be discussed.**

Response: We appreciate your comments. As you have pointed out, we have discussed the limitations of our data in relation to timing exposure (p.15 l.301-303).

Revise: In addition, because we could not determine the timing of exposure, we cannot differentiate between spermatid effects, which we hypothesized, and spermatogonial effects.

**p.15 l.293 – p.16 l.294**

**The sentence is not terribly clear, and the authors should also highlight the role that confounding by some unknown factor may play. So perhaps rephrase as: “Finally, although the results were highly statistically significant, they are based on a quite small sample, and the play of chance, or some unidentified confounding factor, cannot be ruled out.”.**

Response: Per your comment, we have rephrased this for clarity (p.16 l.312-314).

Revise: Finally, although the results were highly statistically significant, they are based on a quite small sample, and the play of chance, or some unidentified confounding factor, cannot be ruled out.

**p.16 l.303-304**

**I would replace the final sentence by “Confirmatory evidence is needed from larger studies which measure the pre-conceptional doses accumulated in various temporal periods, separating out spermatogonial and spermatid effects.”.**

Response: Thank you for this suggestion. We have accordingly revised this (p.16 l.322-324).

Revise: Confirmatory evidence is needed from larger studies which measure the pre-conceptional doses accumulated in various temporal periods, separating out spermatogonial and spermatid effects.
